# Use of systems thinking and adapted group model building methods to understand patterns of technology use among older adults with type 1 diabetes: a preliminary process evaluation

**DOI:** 10.1186/s12874-024-02252-z

**Published:** 2024-06-03

**Authors:** Anna R. Kahkoska, Cambray Smith, Laura A. Young, Kristen Hassmiller Lich

**Affiliations:** 1 Department of Nutrition, University of North Carolina at Chapel Hill, 2205A McGavran Greenberg Hall, Chapel Hill, NC 27599 USA; 2 Division of Endocrinology and Metabolism, University of North Carolina at Chapel Hill, Chapel Hill, NC USA; 3 Center for Aging and Health, School of Medicine, University of North Carolina, Chapel Hill, NC USA; 4 Department of Health Policy and Management, University of North Carolina at Chapel Hill, Chapel Hill, NC USA

**Keywords:** Older adults, Participatory systems science, Continuous glucose monitoring, Systems thinking, Group model building, Diabetes, Qualitative research

## Abstract

**Background:**

A growing number of older adults (ages 65+) live with Type 1 diabetes. Simultaneously, technologies such as continuous glucose monitoring (CGM) have become standard of care. There is thus a need to understand better the complex dynamics that promote use of CGM (and other care innovations) over time in this age group. Our aim was to adapt methods from systems thinking, specifically a participatory approach to system dynamics modeling called group model building (GMB), to model the complex experiences that may underlie different trajectories of CGM use among this population. Herein, we report on the feasibility, strengths, and limitations of this methodology.

**Methods:**

We conducted a series of GMB workshops and validation interviews to collect data in the form of questionnaires, diagrams, and recordings of group discussion. Data were integrated into a conceptual diagram of the “system” of factors associated with uptake and use of CGM over time. We evaluate the feasibility of each aspect of the study, including the teaching of systems thinking to older adult participants. We collected participant feedback on positive aspects of their experiences and areas for improvement.

**Results:**

We completed nine GMB workshops with older adults and their caregivers (*N* = 33). Each three-hour in-person workshop comprised: (1) questionnaires; (2) the GMB session, including both didactic components and structured activities; and (3) a brief focus group discussion. Within the GMB session, individual drawing activities proved to be the most challenging for participants, while group activities and discussion of relevant dynamics over time for illustrative (i.e., realistic but not real) patients yielded rich engagement and sufficient information for system diagramming. Study participants liked the opportunity to share experiences with peers, learning and enhancing their knowledge, peer support, age-specific discussions, the workshop pace and structure, and the systems thinking framework. Participants gave mixed feedback on the workshop duration.

**Conclusions:**

The study demonstrates preliminary feasibility, acceptability, and the value of GMB for engaging older adults about key determinants of complex health behaviors over time. To our knowledge, few studies have extended participatory systems science methods to older adult stakeholders. Future studies may utilize this methodology to inform novel approaches for supporting health across the lifespan.

**Supplementary Information:**

The online version contains supplementary material available at 10.1186/s12874-024-02252-z.

## Background

Type 1 diabetes is a chronic disease in which the pancreas no longer produces insulin, the hormone critical for blood glucose homeostasis [1]. Exposure to elevated blood glucose levels (i.e., hyperglycemia) over time is associated with the development of multiple chronic complications, including neuropathy, retinopathy, nephropathy, and cardiovascular disease, while episodes of low blood sugar (i.e., hypoglycemia) can be life-threatening and require urgent attention [1, 2]. As a result, constant self-management is required to maintain blood glucose levels as near-normal as possible. Unfortunately, self-management is made challenging by dynamic insulin needs, which can be influenced by dietary intake, physical activity, stress, and illness — and thus vary hour-to-hour, day-to-day, and over longer arcs of time impacting health in important ways [2]. As a result, individuals living with Type 1 diabetes are tasked with regularly measuring their blood glucose levels, assessing and accounting for dietary intake, dosing and timing exogenous insulin delivered through injection or insulin pump modalities, responding to hyper- and hypoglycemia, and accounting for other factors such as physical activity, stress, and illness [2].

### Older adults with type 1 diabetes

As the US older adult population (≥ 65 years) grows and the life expectancy associated with a diagnosis of Type 1 diabetes increases, a sizable population of older adults living with Type 1 diabetes has emerged; this population is expected to continue expanding in upcoming years [3]. From a clinical perspective, care and management of Type 1 diabetes in older adulthood is often complex, as patients vary according to age, functional health, presence of frailty, and comorbidity profiles [4]. Compared to younger adults living with Type 1 diabetes for whom the primary focus of care and self-management is on robust glucose control, older adults living with Type 1 diabetes should primarily be focusing on the avoidance of hypoglycemia. Older adults have an increased risk for hypoglycemia, which remains a grave clinical concern due to high morbidity and mortality [4–6]. In addition to ensuring patient safety through the avoidance of hypoglycemia, accommodating patient preferences and preserving quality of life have been outlined as objectives for care [7–10]. However, more specific and applied data to guide care in this population are currently scant, largely owing to relatively recent expansion of this patient population [4, 5, 10].

### Technologic approaches for type 1 diabetes management

New technologic approaches for both glucose monitoring and insulin delivery have been developed to improve strategies for Type 1 diabetes management [2]. One such development is continuous glucose monitoring (CGM), a remote monitoring approach to blood glucose measurement. CGM systems include three components: an on-body sensor with a subcutaneous catheter to measure interstitial glucose approximately every five minutes, a Bluetooth “transmitter,” and an external receiver that displays the real-time blood glucose [11, 12]. CGM is offered currently in two forms, including real-time CGM, or systems that measure and display real-time or near real-time glucose levels at all time, and intermittently scanned CGM, systems that require individuals to scan their device against the sensor to access glucose information [12]. Both types of CGM offer several major advantages over alternative, invasive self-monitoring approaches for blood glucose, which require individuals to frequently obtain a small blood sample *via* finger prick and use a glucometer to measure glucose levels therein. These advantages include access to real-time or near-real time blood glucose information, data on glucose trends (including the rate of rising and falling glucose levels), and less invasive testing methods. Based on growing evidence to suggest clinical and patient-oriented benefits of CGM use, including improved glycemic control and psychosocial wellbeing, clinical practice guidelines now suggest that CGM be offered for all adults with Type 1 diabetes [12]. Practice guidelines specify that adults with diabetes must be capable of using CGM themselves, which may include help from a caregiver, and the specific selection of device should reflect individual patient circumstances, preferences, and clinical needs [12].

### Benefits and challenges of continuous glucose monitoring for older adults with type 1 diabetes

Despite the advantages, a major knowledge gap exists regarding how older adults with Type 1 diabetes interact with, and may ultimately benefit from, diabetes technology like CGM. This gap was highlighted as a critical area for future research in a 2020 consensus statement published on behalf of the International Geriatric Diabetes Society [4]. Data from efficacy-based studies suggest that CGM may confer a significant safety benefit for this age group; in a randomized control trial, use of CGM modestly reduced hypoglycemia over six months among older adults with T1D [13]. The trial measured the duration of hypoglycemia, or the time that blood glucose levels were below 70 mg/dL [13]. Importantly, the reduction in hypoglycemia occurred concurrently with improvements in overall glycemic control, as measured by hemoglobin A1c as well as the time-in-range, or duration of time that blood glucose levels were measured between 70 mg/dL and 180 mg/dL [13]. This finding was important in showing that the reduction in hypoglycemia did not come at the cost of more time spent in hyperglycemic ranges. A handful of observational studies have further reinforced positive effects of CGM in older adults, including decreased hypoglycemia [14], reduced hemoglobin A1c and glycemic variability [15], and increased well-being and feelings of security [16].

Although estimates of the prevalence of CGM use in real-world populations of older adults vary, they range between approximately 30–70% in various studies and settings, suggesting opportunities to increase uptake [14, 17, 18]. It is further established that the general use of medical technology may represent a complicated issue for older adults, particularly with regards to unique, age-specific barriers and the range of biopsychosocial needs that exist across the population [19]. For example, physical symptoms, functional limitations, barriers to care, and psychosocial wellbeing all impact on disease self-management and may impact technology uptake. The growing number of chronic medical conditions accrued in older adulthood lends further complexity to integrating tools that may help improve quality of life. Accessibility features are lacking, including those to address changes to dexterity, visual acuity, and hearing loss [19]. From a psychosocial perspective, older adults may find learning new technologies to be challenging [14] and may require more time for education and training to use CGM and learn to interpret data [17]. Compared to younger adults, studies using questionnaire data have shown older adults perceive substantially higher burdens of technology such as CGM, including concerns that sensor readings cannot be trusted, information from CGM may cause too much worry, and that the technology will be too hard to understand [17]. Interestingly, differences in perceived burdens were substantially less pronounced across age groups in those who use CGM, suggesting that with adequate time, training, and support, older adults can use CGM effectively and experience clinical benefits [13, 15, 17]. However, complex interventions such as this are often plagued with challenges [20], and so identifying the most critical elements to support success as efficiently as possible will increase the likelihood of successful translation across the broader population.

### Objective of the study

There are very limited data on what supports uptake and sustained use of CGM from the perspective of patients and their caregivers, how this technology impacts disease self-management, lived experiences, and clinical outcomes, and what suboptimal responses to technologic approaches over time may occur and why [4, 21]. Our objective thus was to understand the complex nature of older adults’ experiences associated with initiating and sustaining use of CGM, including changes in different clinical, behavioral, and psychosocial variables over time, and how these variables interact to ultimately produce patterns of effective use versus less effective use or nonuse.

These data are needed to inform how clinical recommendations and supports can be developed to help all individuals with Type 1 diabetes incorporate the ever-evolving technologic aspects of diabetes management into their care regimes, regardless of biologic, clinical, and psychosocial differences [22]. As the population of older adults with diabetes grows, these data are also needed to ensure existing and emerging diabetes technology remain accessible across the lifespan.

### Selection of the research methodology

We applied concepts and methods from systems science, specifically Group Model Building (GMB) – a stakeholder-engaged approach to systems thinking from the system dynamics perspective. Key terms relevant for systems thinking and system dynamics are shown in Table 1 along with their definitions.

**Table 1:** Key concepts for systems thinking and system dynamics

Concept	Definition
System	A set of interconnected elements that interact with one another to produce emergent effects distinct from those of its individual components [23, 24]
Dynamic complexity	Behavior exhibited by complex systems with multiple interconnected components, non-linear relationships, time delays, and feedback loops [25, 26]
Feedback loops	Closed chains of causal connections, which can be reinforcing (i.e. when change in an included factor cause a series of changes that ultimately loop back to drive further change in that factor) or balancing (i.e. when change in an included factor cause a series of changes that ultimately loop back to counteract the effects of the initial change); these may have variable time delays [23, 24]
Systems thinking	A cognitive approach arising from multiple disciplines that emphasizes “interconnections, the understanding of dynamic behavior, systems structure as a cause of that behavior, and the idea of seeing systems as wholes rather than parts” [27]
Systems science	An interdisciplinary field that encompasses qualitative and quantitative methods to study the structure, behavior, and dynamics of complex systems [27]
System Dynamics	A subfield of systems science that employs graphical and mathematical models to represent and study the structure, feedback loops, and dynamics of complex systems [28, 29]
Group model building (GMB)	A participatory approach to system dynamics in which diverse stakeholders share and integrate their perceptions and experiences to structure underlying observed behaviors of a dynamically complex system in which improvement is sought [30]
Behavior-over-time graphs	Graphs that focus on patterns of change over time, rather than on an isolated event or outcome, to help people and researchers think about how and why these changes are happening [31]
Reference modes	Real-world patterns of system behavior over time that can be “referred to” (i.e., explained) as part of systems thinking exercises [31]
Causal loop diagramming	A conceptual model that visually represents the hypothesized set of factors, their relationships, and feedback loops that underlie observed or desired trends (reference modes) [32]

The rationale for this approach is as follows. We hypothesized that a complex *system* of factors may shape older adults’ experiences with diabetes self-management and technology use over time, and that a scientific approach to capture *dynamic complexity* in these experiences may offer insights into future interventions. A complex system is a set of interconnected elements that interact with each other to produce emergent effects or collective behaviors that is distinct from the behavior of any of the subcomponents in isolation [24]. These effects persist over time and adapt to changing circumstances [32]. In the setting of technology use, the system could include factors such as physical symptoms or clinical outcomes, lifestyle and behavioral aspects of disease management, wellbeing and psychosocial changes, as well as individual preferences, social and environmental forces, and healthcare resources. Systems science offers methods that can model the structure and complex dynamics of systems (here, those affecting CGM use), while simultaneously looking for direct mechanisms between variables and important points of intervention [33, 34]. Dynamic complexity is an emergent behavior of complex systems, and refers to situations in which effects over time are not easily explained through simple cause and effect, but rather represent the influences from multiple interacting factors that may be non-linear, occur over variable durations of times, and trigger powerful feedback loops to reinforce or counteract earlier changes within the web of interconnections [25, 26, 35].

We specifically aimed to generate a conceptual model of the larger hypothesized system of factors that interact to shape CGM use trajectories (and individuals’ embedded experiences) over time. The model can serve as a way to visualize key pathways where effective technology use and self-management break down, elucidate the problematic outcome trajectories and the constraints of real-life care and support systems, and identify opportunities for change that are aligned with individuals’ experienced system structure.

We thus explored how a participatory (i.e., method that engages stakeholders such as patients and caregivers) system dynamics method called GMB could be leveraged to understand the factors, feedback loops, and system changes that most affect CGM use over time [33, 34]. GMB is a participatory approach to System Dynamics in which diverse stakeholders can exchange their perceptions and experiences to collectively consider the causes of a dynamically complex problem [30, 35–37]. To our knowledge, no GMB studies have yet been published that adapt these methods to specifically engage older adults in improving clinical care by developing a better understanding of broad forces affecting their interactions with evidence-based medical technologies and clinical outcomes.

## Materials and methods

### Study aims

We sought to apply GMB methods to collect data from older adults living with Type 1 diabetes and their caregivers, with considerations to accommodate logistical constraints (e.g., welcoming participants bringing heterogeneous clinical, personal, and professional backgrounds, and limiting the study duration to no more than three hours initially). With this approach, we aimed to bring a systems thinking framework and system dynamics techniques to represent and model the complex processes and outcomes of older adults initiating and using CGM over time and to uncover factors relating to sustained and effective use in daily life. As part of this study, we therefore also explored how systems thinking could be taught to older adult research participants.

### Study design

We developed a facilitation guide and applied it within a series of small (*n* = 3–8), parallel GMB workshops to understand perspectives of older adults with Type 1 diabetes initiating and using CGM over time. The study included two main components: a series of three-hour, in-person, small-group GMB workshops and an optional follow-up series of one-on-one virtual validation interviews. Upon completion of the study, all participants received a $100 (USD) incentive for their time and effort. All study procedures were approved by the Institutional Review Board at the University of North Carolina at Chapel Hill (IRB Study # 21-2331). Participants provided written informed consent prior to participating in the study.

### Study participants

#### Eligibility criteria

For the in-person workshops, patient participants were eligible if: they had a diagnosis of Type 1 diabetes documented in the electronic medical record, were ≥ 65 years of age at the time of recruitment, used an insulin regimen of pump or multiple daily injections, were able to manage their diabetes independently or with the help of a caregiver, had a Hemoglobin A1c level measured within the past year of ≤ 10.0%, and comprehended written and spoken English. Patients were eligible to participate regardless of CGM use. Participants were ineligible if they had a significant medical or psychiatric condition that may have prohibited completion of the workshop, a clinical diagnosis of dementia, or were not fully vaccinated against COVID-19 at the time of recruitment (decreasing risk of transmission within in-person sessions during the pandemic). All potential patient participants were invited to bring a caregiver with them to the research study. Caregiver participants were eligible for the study if they were invited by participants living with diabetes and serve a ‘caregiver’ role in the sense that they provide daily or regular care or support with regards to specific aspects of care or daily management for an older adult (≥ 65 years) with Type 1 diabetes.

#### Recruitment

Study recruitment spanned November 10, 2022–December 13, 2022. Patients were recruited from a single outpatient diabetes clinic at an academic medical center. For the in-person workshops, potentially eligible participants were identified via the electronic health record system and contacted *via* email and telephone outreach. All interested participants were ultimately contacted by telephone following a standardized recruitment script in which participants were provided information about the study and invited to optionally bring a caregiver to the workshop. CGM use status and vaccination status were determined by chart review and confirmed verbally. Participants were scheduled for a workshop on a rolling basis and provided with a series of confirmation and reminder emails.

All participants of the in-person workshop were invited to participate in optional validation interviews; they indicated their preference in writing at the close of the workshop and provided an email address for further contact/questions. There were no incentives offered for the optional validation interview.

### Group model building procedures

Each in-person workshop followed a uniform structure including: (1) completion of a brief questionnaire; (2) the GMB session, including both didactic components and structured activities; and (3) a brief focus group discussion. The workshop lasted three hours, with our agenda shown in Table 2.

**Table 2:** Sample group model building workshop agenda. The agenda included the following note for study participants: This is an interactive workshop where we will essentially brainstorm on the wide range of experiences that older adults may have, as well as the different underlying factors that might lead to those experiences. The agenda below is a guide, but this is a fluid workshop. We will take extra breaks as necessary

Agenda for Workshop Participants	
Agenda Item	Duration
**Informed Consent and Questionnaires**	**15 min**
**Workshop Opening**	**45 min**
Introductions to People and the Goals of the Workshop Activity #1: Warmup Exercise Introduction to Systems Thinking	
**Brainstorming and Drawing Trends**	**50 min**
Example of Systems Thinking: Jan and Betty’s New Year’s Resolution	
Presentation of the Reference Modes: Four Common Patterns of CGM Use in Older Adults	
Activity #2: Drawing and Reflection to Describe Personal Experiences with CGM	
**Break**	**10 min**
**Group Brainstorming**	**40 min**
Activity #3: Using Systems Thinking to Understand Older Adults’ Experiences with CGM	
**Final Reflections**	**20 min**
Brief Focus Group Discussion	
Collect Materials and Distribute Gift Cards	
**Total Time**	**3 h (180 min)**

Each workshop was facilitated by two people, which always included the project lead/first author supported by a second facilitator (co-author or research assistant). The workshop was held in a moderate-sized conference room in an outpatient clinical care site with a designated meeting space for clinical research. The room included a large table, up to 12 chairs, a projector with HDMI connector cables, a screen at the front of the room, and ample wall space for posting study materials adhered with blue painters tape (i.e., wall-safe adhesive). Each study participant was provided with an assigned seat and a clipboard that contained the consent form and HIPAA authorization, the questionnaire, and the workshop packet. Each participant’s seat at the table was marked with a name tag, and there were multiple black and colored pens, two individual whiteboards and colored markers, and two small pads of sticky notes. Due to COVID-19 and the need to maintain masking, no beverages or food were provided, although participants were encouraged to take breaks to eat and drink as needed.

The workshop opening included open-ended prompts for introductions, an icebreaker, and sufficient time for study participants to interact and build rapport before we began the structured aspects of the workshop so that participants would feel comfortable sharing their views and brainstorming in a group. Following brief introductions of the research team, participants were asked sequentially to introduce themselves and describe, to the extent they were comfortable, their relationship to Type 1 diabetes. The introduction prompt was selected to allow for a range of possible responses, which may include narratives surrounding diagnosis, experiences with changing treatment regimens or self-management, attitudes towards Type 1 diabetes, and experiences as caregivers. A separate, informal icebreaker was chosen for each workshop, including, “What is your favorite Thanksgiving food?” and “What is your favorite ice cream flavor?” Although icebreakers are not a requisite aspect of GMB, providing participants with an open structure to tell their stories and allowing time for reactions from other participants early in the workshop aimed to facilitate group bonding to support the rest of the workshop activities.

#### Didactic component

**Fig. 1:**
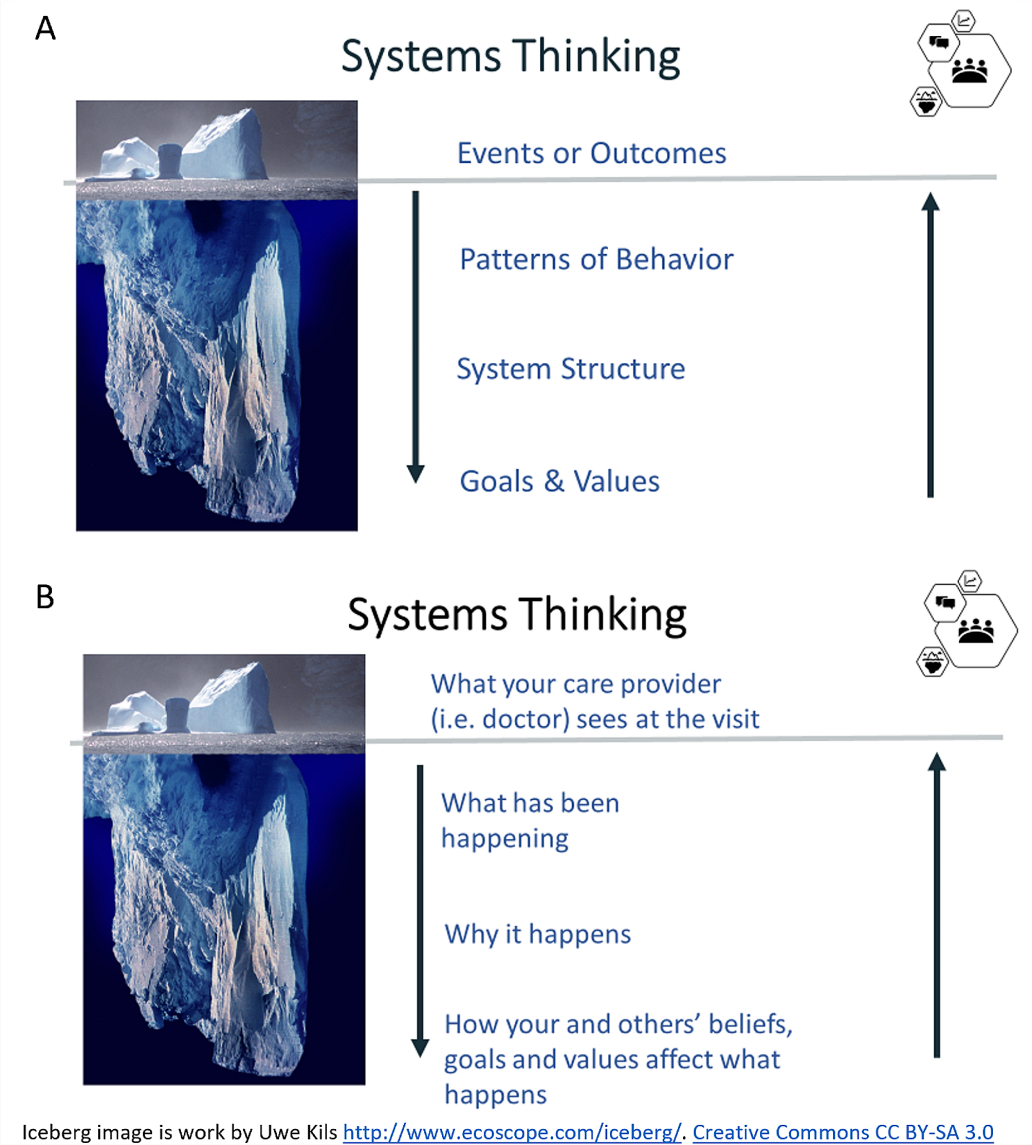
Didactic study components used to present systems thinking. Panel **A** shows the general framework, while Panel **B** shows its extension to understand Type 1 diabetes self-management experiences (**B**). The goal is to work down the iceberg to understand why events/outcomes are happening, and then to use this understanding to identify changes from the bottom up (i.e., in goals, values, and system structure) capable of improving outcomes and trends. Note: the ‘Iceberg Model’ [38] is a widely used approach for teaching introductory systems thinking [39]. The iceberg image is work by Uwe Kils. http://www.ecoscope.com/iceberg/. Creative Commons CC BY-SA 3.0

Each workshop opened with a short presentation by the facilitator that included an overview of the rationale for the study, the goals for data collection, and a series of “ground rules.” The ground rules encouraged participants to share their thoughts freely (and to listen to others respectfully), to draw upon their experiences as the ‘experts’ in the room, and to take breaks as needed. An iceberg metaphor was used to introduce the concept of systems thinking [38], in which isolated events were framed as the ‘tip’ of the iceberg, while related and concerning trends (e.g., root causes,) and problematic aspects of underlying system structure and mental models were reflected as the part of the iceberg that was below the waterline (Fig. 1). Of note, the “Iceberg Model” represents a commonly used image to teach systems thinking by linking events to patterns of system behavior to underlying system structures and mental models [39].

The iceberg metaphor was then extended from a general framework to apply to Type 1 diabetes self-management (Fig. 1B), in which participants were invited to help the research team understand the experiences that happen “below the waterline” as it relates to initiating and using CGM.

The facilitator provided an example of how the systems thinking framework would be applied, which focused on two hypothetical older adult characters in a relatable but distinct example, with the goals of making the method feel practical but not locking thinking into only what is presented in the example. In the example, the characters were friends who set the same New Year’s Resolution to walk 10,000 steps per day and had different outcomes over the following six months. The example was used to introduce two key GMB concepts, including drawing, discussing, and analyzing graphs of system behavior over time, as well as reference modes, which are depictions of real-world patterns of behavior over time that can be “referred to” (i.e., explained) as part of systems thinking exercises [31].

The example and didactic language are presented in full in Appendix A: Primer to Systems Thinking and Systems Mapping.

#### Reference modes

**Fig. 2:**
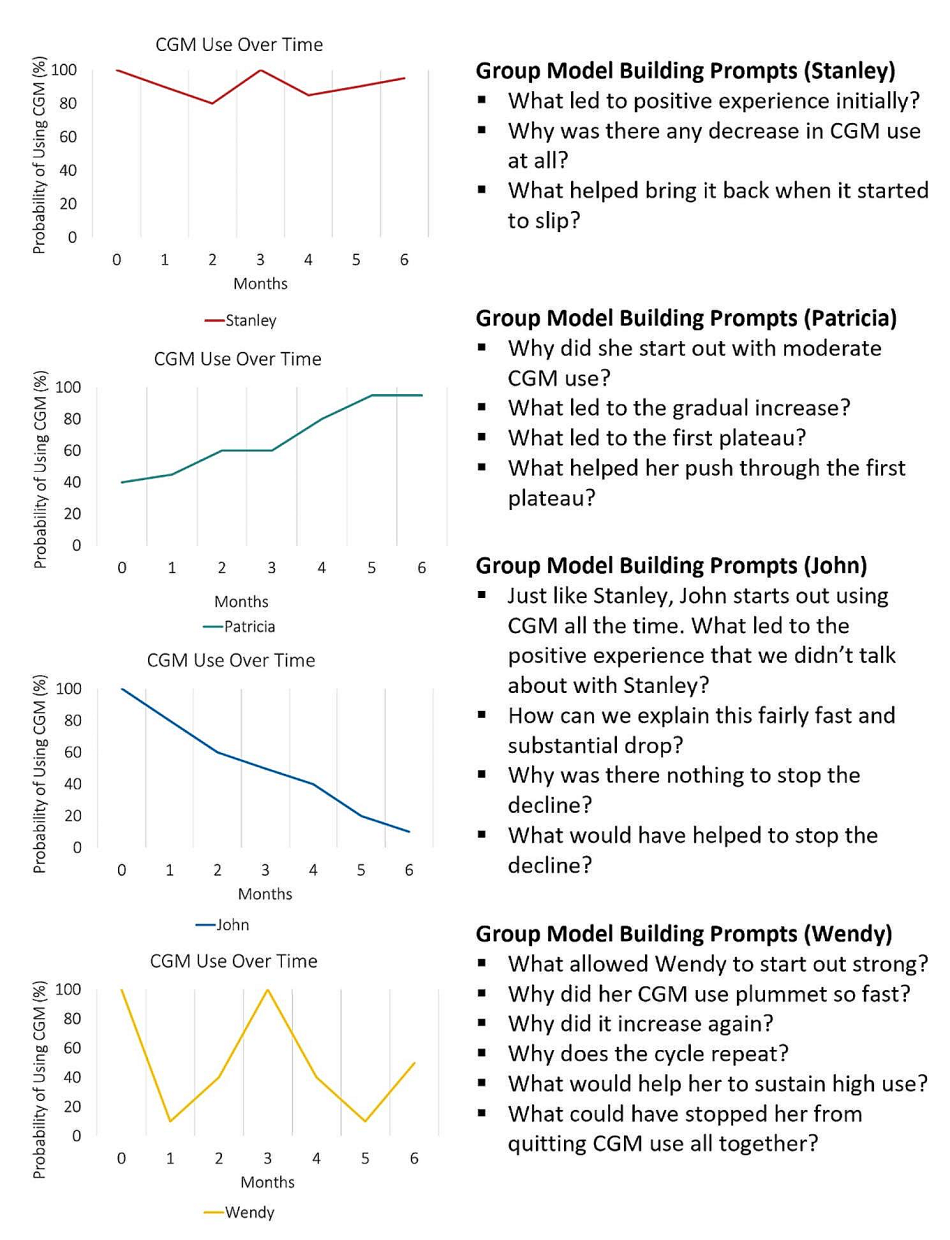
Reference modes provided during the group model building workshop. The reference modes were presented as named characters representing older adults living with Type 1 diabetes who began using CGM as part of their diabetes management. The graphs show the probability of CGM use, defined as both wearing the monitor and using glucose information for diabetes management, over the first six months after CGM is introduced. Four reference modes were selected, including one to represent consistently high use (Stanley), moderate use increasing to high use (Patricia), continually declining use (John), and intermittent use (Wendy)

Following the example of systems thinking, the rest of the workshop focused on CGM use in older adults. We selected four reference modes, or real-world patterns of behavior over time, to reflect common trajectories of optimal and suboptimal CGM use over the first six months following initiation of therapy (i.e., consistently high use, moderate use increasing to high use, continually declining use, and intermittent use/oscillation). We aimed to present sufficiently different reference modes to capture the breadth of common real-world use patterns, while avoiding excessive or redundant trends that may contribute to participant fatigue (i.e., we strived to illustrate the smallest set of distinct reference mode shapes that would elucidate the breadth of qualitatively distinct feedback structures). Each reference mode was presented as a hypothetical older adult character ”persona,” which were used to introduce a 6-month behavior-over-time graph of CGM use (see Fig. 2). We defined CGM use (i.e., Y-axis) as both wearing the CGM and using the readings to make decisions for Type 1 diabetes management, such as ingesting carbohydrates or dosing insulin. Throughout the study, the research team referred to the reference modes by the name of the corresponding older adult character – with the goal of understanding each common behavior-over-time profile. For each reference mode, we strove to draw out stories about key feedback loops operating at different points of time as described in Fig. 2 (e.g., the reinforcing loop that might drive use up or down; balancing loops that slow change – either limiting improvement or counteracting undesired drops in use).

#### Behavior-over-time graphs

Following presentation of the reference modes, the workshop transitioned to drawing and group discussion activities. Behavior-over-time graphs were presented as graphs that focus on patterns of change over time, rather than on an isolated event or outcome, to help people and researchers think about how and why these changes are happening (Table 1). The facilitator introduced the concept of related trends, including guidelines for drawing and annotating trends, and suggested trend topics. Guidelines for brainstorming related trends included: (1) there are no right or wrong answers; (2) trends typically represented nouns or something that can increase or decrease over time unambiguously; (3) there is no need for a formal scale or measurement (i.e., it could be numbers/a specified range or a more qualitative range – low to high); and (4) trends can be consequences or causes of CGM use over time. The facilitator presented an example of how to draw a graph over time, carefully labeling the X-axis as “Time,” noting the start time and end time. The Y-axis was labeled with a variable name and scale, and the understood trend(s) was (were) drawn on the graph and annotated (i.e., reasons the trend changed at specific points in time were noted).

Over the course of the pilot study, we experimented with a range of approaches to encourage the drawing of behavior-over-time graphs. In the first four workshops, study participants were invited to use their Workbook Packet or personal whiteboards to draw their own CGM use patterns and related trends. In the latter five workshops, participants were asked to use their Workshop Packet to identify which reference mode best reflected their own CGM use pattern. Former-users were asked to select the graph which represented their experience, while never-users were asked to select their imagined experience. Participants were then asked to identify and draw three emotions and three benefits or challenges that changed over their first six or more months of using CGM. A sample workshop packet from the latter five workshops can be found in Appendix B: Group Model Building Workshop Packet.

In any workshop, the facilitator answered questions, clarified tasks, and encouraged participants to ask for help if they experienced confusion. If participants were unable to draw themselves, members of the research team offered to listen to their stories and draw behavior-over-time graphs on their behalf. Following drawing exercises, the facilitator led a discussion in which each participant was asked to share and ”annotate” (or explain) their drawings through storytelling and to react to other participant’s drawings and stories.

#### Collective annotation of the reference modes

To collect data for causal loop diagrams to model the system structure underlying common CGM use patterns, we applied a facilitated GMB process based on published scripted group exercises [30, 40].

For each reference mode, the facilitator posed a series of open-ended questions meant to uncover key variables, causal linkages, and feedback loops explaining change over time to be represented within the causal loop diagram. Feedback loops are closed chains of causal connections, which can be reinforcing (i.e. when change in a variable triggers a series of changes or ”ripple effects” that ultimately loop back to drive further change in earlier variables) or balancing (i.e. when change in a variable causes a series of changes that ultimately loop back to counteract the effects of the earlier change) [41–44]. While reinforcing feedback loops can cause exponential growth or decline, balancing loops seek equilibrium within systems; feedback loops may have variable time delays [42, 44]. None of these dynamics is innately good or bad; it depends on desired trends. Our probing questions related to the shape of studied reference modes are shown in Fig. 2. The reference modes were displayed on 24-inch x 36-inch laminated posters around the room, and participants’ ideas were scribed onto small sticky notes and used to annotate the diagram. The facilitator highlighted the feedback thinking for all four reference modes. At the point where a feedback chain became closed, the research team checked with the entire group to see if the chain was correct and complete. Throughout, participants were encouraged to brainstorm together and react to ideas across the group.

Throughout data collection, the research team periodically assessed saturation of themes proposed during the collective annotation of the reference mode. Saturation was defined as the point when no new or original themes emerged. Recruitment ended when saturation was achieved and confirmed through one final meeting in which no new themes emerged.

### Other data collection

#### Focus group discussion

Participants were engaged in a brief focus group discussion at the end of the workshop to provide a final opportunity for sharing thoughts about CGM use in older adults. The focus group discussion was guided by the following four questions: (1) We just talked through four examples here today. Can you think of a story of CGM use over time (i.e., a new reference mode) that we haven’t talked about? (2) With all of this in mind, what do you think are the top three things that we should know, study, or change to help older adults have positive experiences using CGM? (3) When you think about the things you do to take care of your diabetes every day, what are the ways that CGM can help the most? (4) What are expectations and goals that caregivers, doctors, and other members of the care team could have that would be supportive for older adults when they use CGM?

#### Feedback on the research study

At the close of the workshop, participants were asked to use their Workshop Packets to provide feedback on the GMB session to understand how the group model building methodology was perceived among older adults with Type 1 diabetes. Participants were asked to rate their comfort level sharing all their experiences and thoughts (Likert scale; 1–5) and offered an opportunity to share in writing anything additional with the research team that they did not feel comfortable sharing with the group. Participants were also provided space to indicate what they liked and did not like about the workshop. Finally, they were provided with a brief ‘primer’ on systems thinking for optional take-home reading, which reinforced didactic content from the workshop and included additional information about causal loop diagrams (see Appendix A: Primer to Systems Thinking and Systems Mapping.).

Workshop packets were collected and scanned. Photography was used to capture individual and group drawings that occurred outside of the packets, as well as the collective annotations of the reference modes. Workshops were audio-recorded and transcribed. All data were de-identified for analysis.

### Causal loop diagramming

Given overlap in variables generated through GMB across the four reference modes, the research team consolidated and merged data from each reference mode into one collective causal loop diagram depicting the factors, experiences, outcomes, and events that may interact to drive optimal versus suboptimal CGM use patterns over time (Table 1; [45]). As our goal was understanding lived experiences relating to CGM use, we established a system boundary as factors intrinsic to a patient, in a patients’ life (home, social, etc.), or their clinical care environments shaping their CGM use. We designed a core structure to capture factors relating to uptake of CGM and ongoing use of CGM, as well as a subset of ‘endogenous’ drivers of CGM use — factors that affect use and are affected by use (i.e., they are contained within feedback loops that also contain CGM use). Often causal linkages emerged across narratives, but operate in different directions (with directions of initial change determining consequences, and ultimately driving increases or decreases in CGM use). The nature of the relationships between variables was indicated by marking polarity on arrows; an “S” indicates the factors move in the same direction (an increase/decrease in the first variable triggers an increase/decrease in the second) whereas an “O” indicates the variables move in opposite directions (an increase/decrease in the first factor triggers a decrease/increase in the second). In cases where participants’ direct language was deemed to be the most accurate representation of a sentiment or concept in the map, in vivo factors were used.

### Validation of the diagram

A key component of stakeholder-engaged systems science involves iterative refinement and updating of system models with new or changing information [32]. As explicit diagramming was not a part of the in-person workshop, we elected to validate our diagram in follow-up interviews. Participants of the in-person workshop were offered the opportunity to review final causal loop diagram components and offer their feedback through an individual, virtual follow-up interview. The objective of this validation scheme was to ensure that diagrams retained fidelity to the raw data and lived experiences of study participants. Because the full causal loop diagram included many variables and feedback loops, different components of the map were validated in detail with different participants. Validation interviews were 30-minutes and followed a standardized script including a brief overview of the objectives of the research study, a narrative overview of main findings, a viewing of the full causal loop diagram, and a “step-by-step” walk-through of the overall diagram structure and one detailed segment of it (i.e., a subset of loops). Feedback was structured around the following questions: (1) *What are your reactions to the full system map (causal loop diagram)?* (2) *What part of the focused diagram resonates most?* (3) *What pieces of the focused diagram are the most important in determining CGM use over time?* (4) *What is missing from the focused diagram that feels as or more important? This may include making changes such as adding factors, removing factors, or drawing new connections between factors.* Participant feedback was scribed. Validation interviews were performed as dyadic interviews when caregivers were also present. The causal loop diagram was revised iteratively over the course of conducting interviews.

*Ethics approval and Informed Consent*: Ethical approval for this study was obtained from the Institutional Review Board at the University of North Carolina at Chapel Hill (IRB Study # 21-2331). Participants provided written informed consent prior to participating in the study.

## Results

We adapted GMB methods, a participatory approach to system dynamics, to model experiences and trajectories of CGM use among older adults with Type 1 diabetes. We completed nine in-person GMB workshops with older adult patients and their caregivers and generated an integrated causal loop diagram. An in-depth description of study participants and the resulting causal loop diagram are provided elsewhere [45].

Herein, we illustrate data collected through each component of the GMB process, as well as other evaluation measures including recruitment outcomes and feedback on the study from participants as a form of process evaluation.

### Recruitment and attendance

Nine workshops were held between November 15, 2022 and December 15, 2022. Each workshop had between two and six participants. A total of 33 older adults and caregivers participated, of which four were caregivers and the rest were individuals living with Type 1 diabetes. The mean age of the sample was 73.3 ± 4.3 year, with a range of 66–85 years. 55% identified as women, 82% identified as non-Hispanic white, and 12% were non-CGM users.

During recruitment, the main reasons cited for lack of interest or inability to participate included competing medical or surgical appointments, conflicts relating to the winter holidays, and non-local temporary or permanent residence. There were two major challenges for recruitment of an adequately diverse sample. First, the majority of eligible participants for the study within the medical center were non-Hispanic White race and ethnicity. The imbalanced recruitment pool was reflected in a study sample that was majority White and non-Hispanic. Second, there was a small number of eligible participants who were not CGM users, resulting in the majority of study participants being active CGM users.

Attendance of the workshop by recruited individuals was relatively high. A total of four participants did not show for their scheduled workshop. One of those participants was rescheduled for a subsequent workshop, two were unable to be rescheduled, and one was not successfully re-contacted.

### Behavior-over-time graphs

**Fig. 3:**
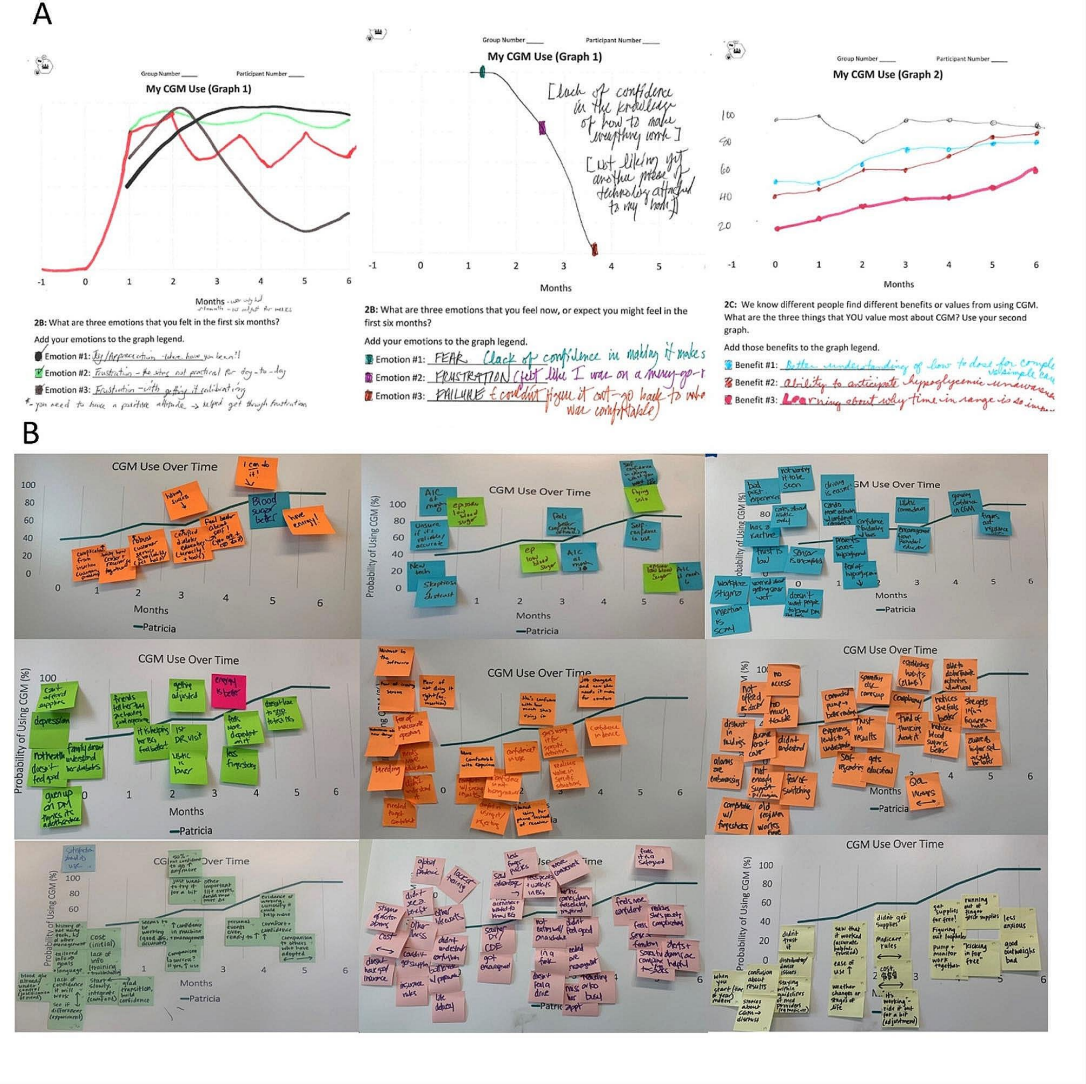
Selected illustrative examples of raw data generated as part of the in-person group model building workshop. Panel **A** shows a subset of individual behavior-over-time graphs drawn by older adults living with Type 1 diabetes and their caregivers. Panel **B** shows the collective annotations of the “moderate use increasing to high use” reference mode

Individual drawing activities proved to be the most challenging aspect of the workshop for study participants, particularly when the drawing prompts were left open-ended. Approaches that facilitated older adults drawing included providing an example of a drawing, pairing a participant with a facilitator to draw on their behalf and in response to their storytelling, and providing more specific prompts, such as asking for graphs of named emotions associated with using CGM or benefits yielded over the first six month. Figure 3 depicts a sample of drawings of emotions and benefits from study participants, including both users and non-users of CGM.

When not all study participants felt comfortable drawing, we found that those who did tended to lead storytelling, which revealed complex dynamics, catalyzing rich group discussions that were captured in the study transcripts and coded for inclusion in the final causal loop diagram. These discussions would draw other participants into the discussion, and also contributed to a significant amount of group bonding, particularly when participants found resonance in their drawings or stories.

### Collective annotation of the reference modes

Workshop participants were successfully engaged through a series of discussion questions to elicit information necessary for causal loop diagramming. Figure 3B shows an example of the raw data produced by collective annotation of the reference modes. Although some themes were constant across groups, different groups generated unique collective ideas, resulting in rich heterogeneity in the annotations across groups. Saturation was achieved in the themes of group annotations by the eighth workshop and confirmed through completion of the ninth workshop.

### Study feedback

Participants expressed that they felt comfortable sharing their thoughts and experiences in the group format, where the mean comfort level was rated as 4.97 on a scale of 1–5 (5 is the highest; *n* = 26 respondents), citing open discussion, a safe and/or welcoming environment, and a clear explanation of how the data will be used as the main reasons for comfort. Several participants expressed value in creating dedicated space for older adults to discuss age-specific aspects of Type 1 diabetes management: “There is great value in listening to the not so good outcomes as we old ducks struggle with the technology…. How best to supply thoughtful, personal support to overcome hesitancy and get folks to actually embrace the technology [Workshop 1, Participant 2].” One participant additionally shared with the research team, “Having the experiences of others was most helpful. Being diabetic sometimes makes you feel alone. Getting to share with your age group was therapeutic [Workshop 3, Participant 4].” Other participant feedback on the GMB workshop is summarized in Table 3, in which participants indicated they liked sharing information and learning from others, finding peer support through shared experiences, the structure and pace of the workshop including small group sessions, age-specific discussions, the systems thinking framework, and the opportunity to contribute to research. Key aspects of feedback for future sessions focused on the duration of the session (where some participants indicated they would like a longer session and other indicated preference for a shorter session) and the lack of food or beverages provided by the research team.

**Table 3:** Illustrative feedback on the in-person group model building workshop from older adults living with Type 1 diabetes and their caregivers

Feedback Theme	Illustrative Response [Workshop #, Participant #]
Things Liked about the Study*	
Opportunity to share experiences with peers	“**Sharing** CGM & other T1 experiences with other diabetics” [WS 1, P1] // “Good chance to **share** experiences and learning” [WS 6 P3]
Learning and exchange of knowledge	“I **learned** a lot of valuable information about type 1 diabetes and CGM. I also **learned** I am not alone in some of the problems I encountered with diabetes.” [WS 2 P1]
Peer support	“Hearing about others going through what I go through” [W 5 P2] // “It let me know that that other participants were or have **similar experiences**.” [WS 9 P3]
Workshop pace and structure	“Everything. Expectations were well-addressed and the concepts we discussed were relevant and important. **Liked the flow–** the explanation and the **freedom to discuss all topics**” [W7 P1]// “it was **fast moving**” [W7 P4]// “**brainstorming**” [W5 P2]
Age-specific discussions	“Talking with other diabetics **close to my age**. It was reassuring to hear others talk about feelings and experiences similar to mine” [W7 P2]
Systems thinking framework	“The researchers. **The iceberg.** The way experiences impact positive and negative behavior. The examples. The exploration of why the graphs over time may have changed” [W9 P2] // “Very interesting methodology-technique for a study” [W3 P1]
Contribution to research	“Knowing that our input will (eventually) be put to use in clinical practice” [WS2 P2]
Areas for Improvement**	
Duration of the workshop	Graphs **too long**—I understand reason—but workshop should be two hours [W6 P2] // **Need more time** in discussion [W8 P3] // **Not enough time**. I LOVE learning about ways to live a longer productive life- and to explore ways that will allow me to do so [W7 P1]
Lack of food or beverages provided	It would be nice to have **water or coffee** available to participants [W6 P3]// **no snacks or refreshments** [W5 P4]

*Participants responded to the following prompt: “What did you like about the workshop?”

** Participants responded in writing to the following prompt: “What did you not like about the workshop? Please provide suggestions for ways to improve the workshop.”

Abbreviations: T1 = Type 1 diabetes. WS = Workshop. P = Participant.

### Focus group discussion

The four focus group questions provided an opportunity to collect any last feedback or perspectives from study participants, but in general, did not reveal new themes or dynamics underlying CGM use that had not been identified through the preceding GMB activities. Participants expressed the sentiment that they had already shared their material they felt to be important relating to CGM use and non-use in older adults.

### Validation interviews

27 of the 33 in-person study participants indicated that they were interested in providing feedback on the diagram. Following completion of the causal loop diagram, eight virtual validation interviews were conducted between March and April 2022. Each interview focused on validating a specific component of the diagram, including the core structure (i.e. the central drivers that impacted CGM use among older adults) and the key feedback loops. Revisions made in validation were minor (i.e., modification of variable names and addition of missing components) and included (1) connecting reactions from caregivers to a perceived sense of intrusiveness, (2) an additional variable to indicate that alarm fatigue would be driven by frequency of alarms, and (3) clarifying that improved HbA1c is associated with a sense of prolonging life alongside preventing complications, both of which promote future CGM use.

## Discussion

We applied GMB, a participatory approach to system dynamics modeling, to collect data from older adults with Type 1 diabetes and their caregivers through group workshops and individual validation interviews to learn about their experiences using technology as part of glucose monitoring regimens. Compared to standard approaches such as surveys, interviews, or focus groups, this systems thinking approach is able to capture the complexity of multiple, interconnected variables that are relevant to an older adult’s experience using CGM, including the feedback loops capable of dramatically impacting long-term CGM use by reinforcing or counteracting earlier changes – sometimes for better and other times for worse. Though the systems thinking approach and methodologies may not be familiar to the readership of clinical journals, there is a growing interest in how systems science methods can be applied to population health and clinical research [33, 34]. Given the richness of our findings and the value the approach added above and beyond more typical approaches (e.g., interview, survey, focus group), we aim to disseminate our approach to introducing and using systems thinking and systems science to diagram the diverse, interrelated factors affecting sustained use of evidence-based technologies in older adult populations.

To our knowledge, few studies to date have extended participatory systems science methods to engage older adult patients. Thus, we consider our study to be a substantial contribution to the literature in that it demonstrates feasibility, acceptability, and value of this methodology to engage older adult patients and stakeholders in research relevant to their health and well-being. This is thus a highly novel study in the field of qualitative systems science approaches as it relates to increasing the diversity of patient stakeholders who are included; older adults have historically been excluded from many clinical research projects. It is unclear why there are so few other studies to employ this method among older adults. One possibility is that the cognitive complexity of GMB may not be seen as feasible among older adults. A recent editorial in the *Journal of the American Medical Association* stated that “structural, or institutional, ageism is not only one of the most potent forms of bias that exists today, but also one of the least acknowledged ” [46]. The authors proceed to cite a report from the World Health Organization that underscores the long-standing history of institutional ageism and the ways in which ageism has become normalized across many domains both in and out of healthcare [47]. A key finding of our study, where we explored best practices to teach systems thinking to older adult research participants, was that the didactic component of the study was well-received, and participants expressed positive feedback for the systems thinking framework, with more than 80% indicating interest in further engagement (i.e., providing feedback on the causal loop diagrams produced). By contrast, the most challenging aspect of the study involved strategies to encourage drawing of behavior-over-time graphs to describe personal experiences with CGM. Future studies that apply GMB with older adult stakeholders will continue to shed insight on how the methodology can be made most accessible and best leveraged to elevate older adult voices as part of the clinical literature that informs future interventions for care and self-management.

Several aspects of the GMB study proved to be effective. The single-session, three-hour long workshop was associated with efficient recruitment and high attendance rates. Although we explored GMB in varied group sizes, we found that data collection was optimized at a group size of between four and six participants, as this size allowed for sufficient exchange of ideas but provided enough time for each participant to speak. Presenting an example of the systems thinking approach as part of the didactic component helped to solidify the framework and prepare participants for the activities to come. We intentionally presented an example focused around a potentially stigmatizing lifestyle change—increasing physical activity—to implicitly reinforce the value of GMB for diving deeper into clinical outcomes or trajectories that may be similarly stigmatized in clinical settings. We hoped that by showing the complexity underlying individual health-related decision-making, that participants would feel comfortable exploring the deeper trends that led to “less than ideal” CGM monitoring. We also drew and provided reference modes for the study, rather than asking study participants to brainstorm and co-create them. In the context of our research question, as well as the time-constraints and need to avoid participant fatigue, we found this approach to be effective. Further, personalizing the reference modes through named older adult characters helped to bridge the graphs to storytelling as part of the collective annotation.

A powerful aspect of the in-person workshops involved the ways in which participants and groups bonded over the duration of the study. We found that bonding occurred regardless of differences in demographics or clinical histories and was largely driven by resonance in day-to-day experiences in managing Type 1 diabetes or age-specific changes in older adulthood. The value of this peer support was reflected in study feedback. From a study design perspective, allowing time and space for long-form introductions prior to the structured presentations and activities was critical. Participants were generally eager to exchange tips or resources for Type 1 diabetes management and effective CGM use, although the research team always stressed the importance of talking with a healthcare provider about any clinical changes to their healthcare plan. Several participants expressed interest in continuing to meet with other participants in their workshops to continue conversations and the exchange of information and experiences.

There were several aspects of the study that were more challenging. One of the major limitations was the ability to recruit an adequately diverse sample to reflect the heterogeneity of the population of older adults living with Type 1 diabetes. The limited number of participants from underrepresented racial and ethnic groups (e.g. those who identified as Black and/or Hispanic) and non-CGM users likely reflects a combination of selection bias related to recruiting from a single academic medical center, as well as a degree of survivor bias in which individuals with diabetes who did not have access to high-quality treatment or the ability to self-manage effectively earlier in disease duration were not represented; in cohort studies, excess mortality associated with Type 1 diabetes has been shown to disproportionately affect African American individuals compared to their White counterparts [5]. Given the in-person component of the study, the majority of study participants were also local to the city in which the academic medical center was located. Further, the selection bias associated with the COVID-19 vaccination requirement likely represents a complex medical and social bias, the effect of which on the study findings is difficult to characterize. Future work to include more diverse participants within group sessions is critically needed to ensure that conceptual models and diagrams are valid for this population.

There are other limitations to external generalizability of the findings as our study did not include older adults with cognitive, visual, or hearing impairments, or HbA1c greater than 10%. Future work is needed to engage populations with additional health challenges that may complicate diabetes management, as well as the subpopulations for whom hypo- and hyperglycemia represent major clinical issues. Because GMB focuses on working together to describe stories, having a group that shares the same language is also a requirement, further narrowing our sample and thus the overall representativeness of this study.

Future GMB studies for older adults may consider the following points of learning. First, data collection may be enriched with regards to underrepresented views, including one or more group study sessions dedicated to capturing the experiences of such individuals, without dilution or bias from influence of subgroups that tend to be over-represented [48]. In our study, a small proportion of older adults living with Type 1 diabetes elected to bring a caregiver with them to the study. Future studies which aim to integrate diverse stakeholder perspectives, such as those from caregivers, may consider recruiting these stakeholders independently for participation in a focused workshop of caregiver participants only. Although we included a brief focus group discussion as part of our workshop, we found that participant responses were very brief or largely redundant with data collected through the drawing and annotation exercises; thus, this section of the study did not significantly expound upon or enrich data. We believe this reflects, in large part, the open-ended nature of the preceding GMB activities and vigorous group discussion. Finally, in review of the transcripts of the study, we found that participant narratives yielded significant contextual information, personal narrative, or other ‘foreground’ for clinical questions that are well-suited for inductive qualitative analysis techniques. Our research team aims to explore how the results from system dynamics analyses and varied qualitative analyses [49] can be triangulated to provide a complementary, comprehensive view of the lived experiences of older adults with Type 1 diabetes, as well as their complex experiences with technology.

There are several questions remaining. It remains unclear how best providers can be integrated into GMB, and if the potential for power dynamics between patient and provider stakeholders may influence the quality of GMB in mixed groups. Recruiting patient and provider stakeholders across institutions or holding provider-specific workshops may avoid this problem, although validation between groups may prove more challenging for the latter. It is also unknown how the causal loop diagrams may change if older adult participants are directly involved in the diagraming process, and how the diagrams would change between sessions. Studies that include older adult participants in direct diagraming may need to explore various study formats to ensure that burden remains low, and participants do not experience significant fatigue. Given that the number of feedback loops in our causal loop diagram exceeded 100, it is likely that generating comprehensive diagrams with participants directly may involve more than one workshop, thus increasing the burden of the research study and potentially hampering recruitment outcomes. Alternatively. research teams might explore the potential of having workshops build on earlier diagrams, adding content believed to be as or more important as what was included in prior sessions.

It has been argued that systems thinking and systems science methods, such as system dynamics, remain underutilized for complex health problems [33, 34, 50, 51]. For the study objective, which demands an awareness of more dynamic complexity than traditional research methods [34], GMB provided a novel approach among older adults to comprehensively investigate and describe multiple, interrelated factors that determine the uptake and use of medical technologies, as well as their complex interactions over time. The holistic view of experiences as the behavior of a complex system offers the opportunity to not only describe, but start to untangle the mechanisms that shape older adults’ experiences with technology and how it fits into broader chronic disease self-management [42, 52]. In related work, our team used the causal loop diagrams to identify outcome sets which represent ‘suboptimal CGM responses’ that signal the need for additional resources, education, or support, as well as the system structure of the factors that interact to produce each response; we will use this problem definition as the basis for efforts to develop new strategies to address and prevent suboptimal trajectories associated with CGM use in older adults with Type 1 diabetes [45]. We are also enthusiastic to extend the GMB methodology, and integrate lessons learned in the present study, to continue to engage older adults as primary stakeholders in research to promote the access to and use of medical technology for longevity and healthy aging across a range of clinical contexts.

### Electronic supplementary material

Below is the link to the electronic supplementary material.

Supplementary Material 1

Supplementary Material 2

## Data Availability

The data that support the findings of this study are available upon reasonable request from the corresponding author, ARK. The data are not publicly available due to their containing information that could compromise the privacy of research participants.

## Abbreviations

CGM: Continuous Glucose Monitoring
GMB: Group Model Building
